# Posterior Reversible Encephalopathy Syndrome Induced by Mirtazapine Overdose: A Case Report

**DOI:** 10.1155/crnm/4690032

**Published:** 2025-06-06

**Authors:** Min-Chiao Tsai, Wei-Hao Lin

**Affiliations:** ^1^Department of Psychiatry, National Cheng Kung University Hospital, Tainan, Taiwan; ^2^Department of Neurology, Kaohsiung Medical University Hospital, Kaohsiung, Taiwan

**Keywords:** case report, mirtazapine, posterior reversible encephalopathy syndrome, serotonin syndrome, status epilepticus

## Abstract

**Introduction:** Posterior reversible encephalopathy syndrome (PRES) is a neurological emergency typically associated with hypertension or drug toxicity. Although mirtazapine is not a classical serotonergic agent, overdose may induce serotonin syndrome, which can contribute to PRES.

**Case Presentation:** A 50-year-old woman presented with seizures, impaired consciousness, and autonomic instability following ingestion of > 300 mg mirtazapine. Magnetic resonance image (MRI) revealed vasogenic edema in the parieto-occipital and frontal lobes. Her clinical features fulfilled the Hunter criteria for serotonin syndrome. Treatment with cyproheptadine led to full clinical and radiological recovery.

**Discussion:** Serotonin syndrome may disrupt cerebral autoregulation and impair endothelial integrity, contributing to PRES. Although rare, similar cases have been reported with other serotonergic agents. This is the first reported case of mirtazapine overdose resulting in serotonin syndrome–associated PRES.

**Conclusion:** Clinicians should recognize that mirtazapine overdose can cause serotonin syndrome and secondary PRES. Early identification and serotonin antagonism are crucial for recovery and prevention of sequelae.

## 1. Introduction

Posterior reversible encephalopathy syndrome (PRES) is a neurological emergency characterized by impaired consciousness, blurred vision, headache, or seizures. It is often triggered by underlying conditions, such as malignant hypertension, or by medications that impair endothelial function or cerebral autoregulation, including immunosuppressants and chemotherapeutic agents. The vasogenic edema resulting from endothelial dysfunction and impaired cerebral autoregulation is typically observed in the occipital and posterior parietal lobes [1]. While serotonin syndrome and antidepressants are not commonly recognized as risk factors for PRES, here, we present a case of PRES associated with serotonin syndrome due to a mirtazapine overdose. To our knowledge, this is the first reported case of mirtazapine overdose leading to serotonin syndrome, which subsequently contributed to the development of PRES.

## 2. Case Presentation

A 50-year-old woman was brought to the emergency department after being found unconscious on the floor. Her medical history included opioid and amphetamine use disorders (both in sustained remission), alcohol use disorder, depressive disorder with chronic insomnia treated with mirtazapine 60 mg/day, propranolol 10 mg/day, and zolpidem 20 mg/day, as well as a history of anemia. She was known to have poor medication adherence.

There was no known history of hypertension, renal disease, or connective tissue disease, and no history of exposure to medications such as immunosuppressants, chemotherapeutic agents, or lithium. According to her family, she had last been seen in her normal state approximately 18 h earlier during a phone call.

On arrival, the patient exhibited generalized convulsions, upward gaze, and urinary incontinence. Status epilepticus (SE) was initially suspected. She was treated with intravenous lorazepam 2 mg stat and intravenous lacosamide 200 mg twice daily. Although the seizures resolved, her consciousness remained impaired with a Glasgow Coma Scale (GCS) score of E2V3M3. Her body temperature was 37.8°C, and her blood pressure was 166/106 mmHg. Neurological examination revealed bilaterally dilated pupils (6/6 mm) with sluggish light reflexes, along with tremors, myoclonus, rigidity, and hyperreflexia in all four limbs.

Laboratory investigations revealed normal glucose, electrolytes, thyroid function, liver and renal function, C-reactive protein, and procalcitonin. Serum creatine kinase was elevated at 328 IU/L. Urine drug analysis for morphine and amphetamine was negative. Given the absence of systemic signs of infection and normal infection markers, central nervous system (CNS) infection was considered unlikely, and the patient's family declined cerebrospinal fluid (CSF) analysis.

**Figure 1:**
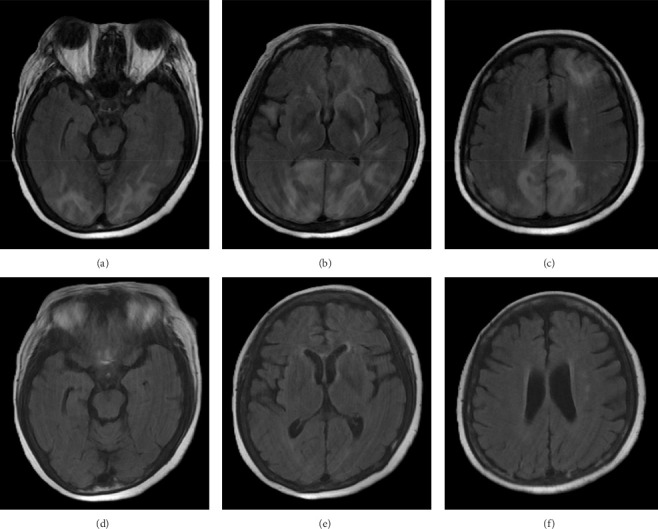
Brain MRI (FLAIR sequences) before and after treatment. (a–c) Axial FLAIR images obtained during the acute episode show hyperintense signals in the bilateral occipital, parietal, and left frontal lobes. Images are arranged from inferior (a) to superior (c). (d–f) Follow-up images acquired 4 months after treatment demonstrate complete resolution of the previously noted abnormalities. Images are arranged from inferior (d) to superior (f). Abbreviations: FLAIR: fluid-attenuated inversion recovery; MRI: magnetic resonance imaging.

A brain magnetic resonance image (MRI) revealed hyperintensities in the bilateral parietal, occipital, and left frontal lobes on fluid-attenuated inversion recovery (FLAIR) sequences (Figures 1(a), 1(b), and 1(c)), suggestive of PRES. Continuous electroencephalogram (EEG) monitoring showed no epileptiform discharges, despite ongoing myoclonus.

Based on the clinical presentation, laboratory data, neuroimaging, and EEG findings, the differential diagnosis included SE, serotonin syndrome, and neuroleptic malignant syndrome. Upon reviewing her medication history, her family reported that the patient had consumed alcohol and ingested over 300 mg of mirtazapine on the night before she was found, during her last known contact, a phone call approximately 18 h earlier. According to the Hunter Serotonin Toxicity Criteria, the patient's presentation, including exposure to a serotonergic agent, myoclonus, tremor, and hyperreflexia, was consistent with serotonin syndrome.

To manage the serotonin syndrome, intravenous lorazepam 2 mg was administered every 8 h, but the clinical response was limited. Cyproheptadine, a serotonin antagonist, was initiated at a dose of 4 mg three times a day. After 3 days of cyproheptadine treatment, the patient's consciousness gradually improved to a GCS score of E4V5M6. Neurological signs, including muscle rigidity, myoclonus, tremors, and mydriasis, also resolved, and her blood pressure returned to the normal range. Based on the clinical progression and the patient's response to cyproheptadine, a final diagnosis of serotonin syndrome–associated PRES was established. This diagnosis was further supported by a follow-up brain MRI performed 4 months later, which demonstrated complete resolution of the previously noted abnormalities (Figures 1(d), 1(e), and 1(f)).

## 3. Discussion

PRES is a condition characterized by both clinical and radiological findings. Common clinical manifestations include impaired consciousness, blurred vision, headache, and seizure, including SE. On MRI, white matter hyperintensities can be observed on FLAIR sequences, typically in the occipital and adjacent posterior parietal lobes. The etiologies of PRES include severe hypertension, its most common underlying cause, as well as eclampsia and medication toxicity. These factors are thought to contribute to cerebrovascular dysregulation or endothelial dysfunction [1].

Serotonin syndrome is a life-threatening neurologic condition caused by excessive serotonergic activity in the CNS. It is most commonly triggered by medications such as antidepressants, antipsychotics, anticonvulsants, antiparkinsonian agents, and antimigraine medications. Symptoms typically develop within 24 h of serotonergic overdose [2]. Diagnosis is guided by the Hunter Serotonin Toxicity Criteria, which require the use of a serotonergic agent plus one of the following clinical features: (1) spontaneous clonus, (2) inducible clonus plus agitation or diaphoresis, (3) ocular clonus plus agitation or diaphoresis, (4) tremor plus hyperreflexia, or (5) hypertonia plus temperature above 38°C and ocular or inducible clonus [3]. In our case, the patient's exposure to a high dose of mirtazapine, along with the presence of tremor, myoclonus, and generalized hyperreflexia, fulfilled the Hunter criteria for serotonin syndrome. Treatment includes immediate discontinuation of serotonergic agents, supportive care, sedation with benzodiazepines, and, when indicated, administration of serotonin antagonists such as cyproheptadine [4].

Mirtazapine indirectly enhances serotonergic transmission by antagonizing 5-HT2 and 5-HT3 receptors, thereby increasing 5-HT1A receptor activity, which may contribute to serotonin syndrome in overdose settings [5, 6]. Ethanol further amplifies serotonergic signaling by elevating extracellular serotonin and impairing its clearance [7]. Although rare with mirtazapine monotherapy, the combination of mirtazapine overdose and alcohol ingestion in this case likely acted synergistically to precipitate serotonin toxicity.

The underlying pathophysiology of PRES remains multifactorial, with impaired cerebral autoregulation and endothelial dysfunction being the two leading hypotheses. In the context of serotonin syndrome, acute hypertension and autonomic instability may disrupt cerebral autoregulation, resulting in cerebrovascular hyperperfusion and subsequent blood–brain barrier breakdown [8]. In addition, serotonergic agents have been reported to transiently alter cerebrovascular tone by interfering with myogenic vasoconstriction and calcium homeostasis in cerebral vascular smooth muscle [9]. These alterations may further compromise autoregulatory mechanisms and contribute to hyperperfusion. Moreover, experimental data suggest that antidepressants at supratherapeutic levels exert direct cytotoxic effects on blood–brain barrier endothelial cells, exacerbating endothelial dysfunction [10]. Taken together, the synergistic effects of impaired autoregulation and endothelial injury in serotonin syndrome likely converge to promote hyperperfusion and vasogenic edema, ultimately leading to PRES.

Although rare, serotonin syndrome–associated PRES has been described. Malik et al. [9] reported a case linked to venlafaxine and buspirone, while Prakash et al. [8] described a similar presentation with amitriptyline and paroxetine. In our case, mirtazapine overdose was the likely precipitant. All three patients met the Hunter criteria for serotonin syndrome and exhibited characteristic MRI findings of vasogenic edema in the parieto-occipital regions. These imaging abnormalities resolved following appropriate treatment, including cyproheptadine.

## 4. Conclusion

This case underscores a potential but underrecognized link between serotonin syndrome and PRES. Although mirtazapine is not a typical serotonergic agent, it can indirectly enhance serotonergic activity via 5-HT1A receptor stimulation, particularly in overdose and when combined with alcohol. Clinicians should be aware of this association, as prompt recognition and treatment of serotonin syndrome, through drug withdrawal and serotonin antagonism, are critical for the resolution of PRES and the prevention of neurological sequelae.

## Data Availability

The data that support the findings of this study are available on request from the corresponding author. The data are not publicly available due to privacy or ethical restrictions.

## Nomenclature

PRES: Posterior reversible encephalopathy syndrome
SE: Status epilepticus
GCS: Glasgow Coma Scale
CNS: Central nervous system
MRI: Magnetic resonance image
FLAIR: Fluid-attenuated inversion recovery
EEG: Electroencephalogram
